# Methicillin-resistant Staphylococci in Companion Animals

**DOI:** 10.3201/eid1112.050241

**Published:** 2005-12

**Authors:** Keith E. Baptiste, Kerry Williams, Nicola J. Willams, Andrew Wattret, Peter D. Clegg, Susan Dawson, John E. Corkill, Turlough O'Neill, C. Anthony Hart

**Affiliations:** * University of Liverpool, Leahurst, United Kingdom; † Royal Liverpool University Hospital, Liverpool, United Kingdom

**Keywords:** MRSA, horses, dogs, veterinary hospitals, zoonosis, dispatch

## Abstract

We determined the molecular characteristics of methicillin-resistant staphylococci from animals and staff at a small animal and equine hospital. Methicillin-resistant *Staphylococcus aureus* (MRSA) identical to human EMRSA-15 was found in dogs and hospital staff. In contrast, 5 distinct MRSA strains were isolated from horses but not from hospital staff.

Methicillin-resistant *Staphylococcus aureus* (MRSA) is among the most important causes of human healthcare associated infections. MRSA has also caused infections in dogs (*1**,**2*), and cases of human-to-dog transmission of MRSA in which dogs have acted as reservoirs for reinfection have been reported (*3**,**4*). MRSA and methicillin-resistant, coagulase-negative staphylococci (MR-CNS) have also been reported in horses (*5*), including outbreaks in equine hospitals (*6*). In these cases, MRSA was thought to be of human origin (*6*); however, a Japanese study could not definitively relate equine to human MRSA with pulsed-field gel electrophoresis (PFGE) (*7*). At a Canadian equine hospital and thoroughbred farm, both horses and staff were positive for MRSA, and 96% and 93% of isolates, respectively, were subtypes of a rare Canadian MRSA-5 clone (*8*).

Horses, dogs, and cats in the community; animals treated at the University of Liverpool's Small Animal Hospital (SAH) and Philip Leverhulme Equine Hospital (PLEH); and staff at those hospitals were screened for MRSA. The molecular characteristics of MRSA in these populations were investigated to determine the source and routes of transmission. Animal samples were also screened for MR-CNS.

## The Study

Swabs were taken from the anterior nares of dogs, horses, and staff; nasal surface of cats; perineum of dogs, cats, and horses; and the neck skin surface of horses. All diagnostic submissions from both of these hospitals were screened for MRSA. Swab specimens were directly inoculated onto mannitol salt agar (LabM, Bury, UK) with aztreonam (2 mg/L) and oxacillin resistance–screening agar (Oxoid, Basingstoke, UK) and incubated at 37°C for <48 h. Staphylococci were identified by colony shape, Gram stain, staphylase test (Oxoid), and API staph kit (MR-CNS only) (bioMérieux, Basingstoke, UK). The disk-diffusion method (Mast, Liverpool, UK) was used to determine the susceptibility of all isolates to oxacillin, methicillin, gentamicin, vancomycin, rifampicin, ciprofloxacin, co-trimoxazole, fusidic acid, and tetracycline, according to the British Society for Antimicrobial Chemotherapy guidelines, by using *S. aureus* ATCC 26923, EMRSA-15, and EMRSA-16 as controls (*9*).

Cell lysates of all methicillin-resistant staphylococci were prepared as described previously (*10*). Cell lysates were also prepared from 3 control strains, EMRSA-15, EMRSA-16, and the Canadian epidemic strain, CMRSA-5, previously found in horses and humans (*8*). The presence of the *mecA* gene was determined with polymerase chain reaction (PCR) by using a modified method adapted from Vanuffel et al. (*11*), with a conventional thermocycler. PCR to detect the *S. aureus femA* gene was used to confirm isolates as MRSA (*12*). For all MRSA and equine MR-CNS isolates, the SCC*mec* cassette and the *agr* operon were analyzed as described previously (*13*). All MRSA isolates were screened for the gene encoding Panton-Valentine leukocidin by using the method of Lina et al. (*14*); a positive control for this reaction was provided by the Scottish MRSA Reference Laboratory. Macrorestriction of the genome and PFGE were conducted on all MRSA isolates according to the protocol described by Murchan et al. (*15*) and included on each gel with EMRSA-15 and -16 and CMRSA-5.

Swabs taken from cats (n = 50) and dogs (n = 55) treated at the SAH and cats within the community (February–March 2004) were negative for MRSA. One cat was positive for methicillin-resistant staphylococci, and 4 dogs were positive for MR-CNS, all of which were confirmed by PCR to be carrying the *mecA* gene. However, 3 dogs with clinical infections (a joint infection in January 2004, pleuropneumonia in March 2004, and a wound infection in June 2004) were positive for MRSA at the site of infection. The dog with the joint infection was also positive for nasal and fecal carriage of MRSA; a student who treated the dog had an MRSA-positive nasal swab in April 2004. Eleven staff provided nasal swabs, of which 2 were positive for MRSA in January 2004 (Table). All MRSA isolates were resistant to ciprofloxacin but sensitive to all other antimicrobial drugs tested. All MRSA isolates were positive for the *mecA* and *femA* genes, carried the SCC*mec* type IV cassette, and were *agr* operon group 1 strains but were negative for *pvl* genes. PFGE showed that the human and dog clinical MRSA isolates were identical to the human epidemic strain, EMRSA-15 (Figure).

**Table:** Isolate test results for methicillin-resistant Staphylococcus aureus (MRSA)*

	No. sampled	No. samples positive for MRSA† (%)			Other (clinical)	No. samples positive for MR-CNS† (%)		
		Nasal	Perineum	Skin				
Dogs								
Clinical cases	3	1	1	1	Joint and pleural fluid, feces	NT	NT	NT
SAH	32	0	0	0		2 (6)	1 (0)	0
Community	22	0	0	0		1 (5)	0	0
Cats								
SAH	26	0	0	0		0	0	0
Community	24	0	0	0		1 (5)	1 (5)	0
SAH veterinary staff	11	3 (27)	NT	NT		NT	NT	NT
Horses								
Clinical cases	3	1	NT	1	Pleural and joint fluid	NT	NT	NT
PLEH	67	8 (12)	0	2 (3)		6 (9)	3 (5)	5 (8)
Community	40	0	0	0		12 (30)	0	1 (3)
PLEH Veterinary staff	12	0	NT	NT		NT	NT	NT

*SAH, small animal hospital; PLEH, Philip Leverhulme Equine Hospital; NT, not tested; MR-CNS, methicillin-resistant, coagulase-negative staphylococci.

†Some animals were positive for >1 body site.

**Figure:**
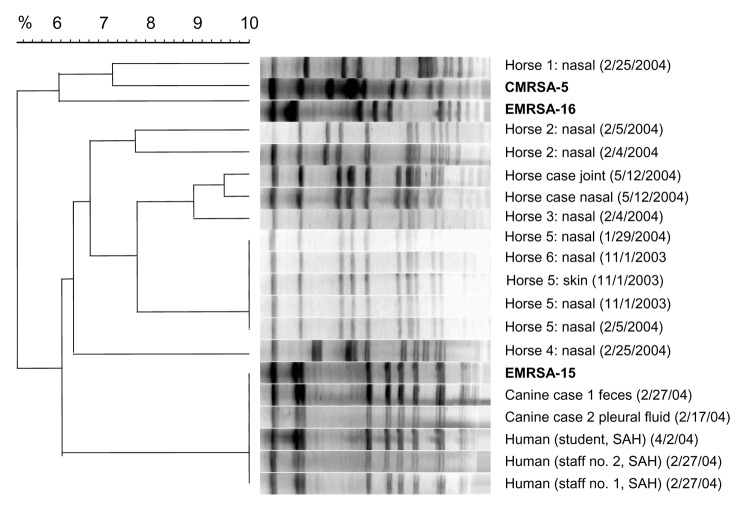
Dendrogram showing the pulsed-field gel electrophoresis patterns after macrorestriction of genomic DNA with SmaI of methicillin-resistant Staphylococcus aureus (MRSA) isolates from the small animal hospital (SAH) and the equine hospital. The dog and human isolates (SAH staff) were identical to the UK major epidemic strain EMRSA-15, and the equine MRSA isolates (5 distinct profiles) were unrelated to EMRSA-15, EMRSA-16, or CMRSA-5. Profiles were analyzed with Molecular Analyst software (Applied Maths, Inc., Sint-Martens-Latem, Belgium) by unweighted pair grouping by mathematical averaging clustering method with a 2% tolerance window and using the Dice coefficient.

Of the 105 horses sampled, MRSA was isolated only from horses at PLEH. Of the 67 horses sampled at PLEH, 11 were positive (16%) for carriage and 3 had MRSA-associated clinical infections (pleuropneumonia, chronic septic arthritis, and chronic dermatitis). None of the isolates submitted from 12 staff members at the equine hospital were positive for MRSA. The horse MRSA isolates were resistant to gentamicin (100%), rifampicin (80%), ciprofloxacin (78%), fusidic acid (69%), co-trimoxazole (50%), and tetracycline (50%) but not to vancomycin. All MRSA isolates were positive for the *mecA* and *femA* genes and were *agr* group 1, except 2 that were *agr* group 2, but all were negative for the *pvl* genes. Like the human and dog isolates, all horse MRSA isolates except 3 (1 isolate had a variant of type II or III, and 2 isolates repeatedly failed to give PCR products for SCC*mec* cassettes), carried the SCC*mec* cassette type IV. MR-CNS was isolated from 19% of horses at the PLEH and 30% of horses in the community. All horse MR-CNS isolates (including those from PLEH) had different SCC*mec* cassettes than the MRSA isolates, and their banding patterns did not fully correspond to any of the known cassette types, giving a 209-bp band (types II and III) and a further band of 495 bp (type I). Twelve MRSA isolates from 7 horses were selected for PFGE based on differences in antibiogram and genes detected by PCR. This analysis showed 5 distinct strains. The same strain found in nasal samples, 1 skin sample from 3 horses, and 1 MRSA strain from a clinical case-patient were closely related to a nasal isolate from a different horse. None of the horse MRSA strains were related to EMRSA-15, EMRSA-16, or CMRSA-5 as demonstrated in the Figure.

## Conclusions

This study documents MRSA transmission between humans and dogs; the same strain was found in 3 staff members and 3 dogs, all identical to the predominant human epidemic strain EMRSA-15. Two staff members and a student who treated 1 dog were positive for the same MRSA strain. Furthermore, MRSA was associated with clinical disease in 2 other dogs some months later; this finding could suggest a cycle of transmission between staff and animals. However, the origin of MRSA in the first dog is unknown and could have originated in either staff or the dog in question, with dog-to-human transmission or vice versa. This study suggests that dogs can act as reservoirs of MRSA, which can pose a public health risk to owners and veterinary staff, as well as limit the options for antimicrobial drug treatment of MRSA infections. Staff in veterinary hospitals could have an increased risk of carrying MRSA because of contact with infected animals and antimicrobial drugs in their work environment.

Contrary to SAH results of this study and previous work in Canada, no evidence was seen of MRSA transmission between staff and horses at PLEH, nor were any isolates related to the predominant UK human epidemic strains or CMRSA-5. However, 5 different horse MRSA strains were identified with unknown sources. The fact that different SCC*mec* cassettes were found in horse MR-CNS isolates than in MRSA isolates does not suggest that methicillin resistance had transferred from MR-CNS to MRSA. Furthermore, the prevalence of MR-CNS in horses in the community is almost double that which was found in horses at PLEH. This could suggest that MR-CNS may compete well with methicillin-sensitive CNS in an environment where antimicrobial drugs are not present. These results imply that MRSA is present in the general horse population and may represent a reservoir of new or rare MRSA strains that could be transmitted to humans.
